# Functional and Structural Signatures of the Anterior Insula are associated with Risk-taking Tendency of Analgesic Decision-making

**DOI:** 10.1038/srep37816

**Published:** 2016-11-28

**Authors:** Chia-Shu Lin, Hsiao-Han Lin, Shih-Yun Wu

**Affiliations:** 1 Department of Dentistry, School of Dentistry, National Yang-Ming University, Taipei, Taiwan (ROC); 2 Division of Family Dentistry, Department of Stomatology, Taipei Veterans General Hospital, Taipei, Taiwan (ROC).

## Abstract

In a medical context, decision-making is associated with complicated assessment of gains, losses and uncertainty of outcomes. We here provide novel evidence about the brain mechanisms underlying decision-making of analgesic treatment. Thirty-six healthy participants were recruited and completed the Analgesic Decision-making Task (ADT), which quantified individual tendency of risk-taking (RPI), as the frequency of choosing a riskier option to relieve pain. All the participants received resting-state (rs) functional magnetic resonance imaging (MRI) and structural MRI. On rs-functional connectome, degree centrality (DC) of the bilateral anterior insula (aINS) was positively correlated with the RPI. The functional connectivity between the aINS, the nucleus accumbens and multiple brain regions, predominantly the medial frontal cortex, was positively correlated with the RPI. On structural signatures, the RPI was positively correlated with grey matter volume at the right aINS, and such an association was mediated by DC of the left aINS. Regression analyses revealed that both DC of the left aINS and participants’ imagined pain relief, as the utility of pain reduction, could predict the individual RPI. The findings suggest that the functional and structural brain signature of the aINS is associated with the individual differences of risk-taking tendency in the context of analgesic decision-making.

When making a medical decision – either for choosing over-the-counter medicines or for shared decision-making between patients and clinicians – one needs to carefully balance between both gains (e.g., therapeutic potency) and losses (e.g., the adverse effect)^1^,^2^. Behavioral findings have revealed that the choice about an analgesic treatment, a very common scenario of medical decision-making^3^,^4^, is influenced by multiple treatment-related attributes, including the potency in pain reduction, the probability that the treatment would work successfully, the probability that an adverse effect would occur, and the time course of the therapeutic effect^5^,^6^. These studies adopted the Analgesic Decision-making Task (ADT), which was designed to mimic the clinical scenarios where one needs to choose between a conservative or ‘riskless’ option was less potent, with a higher probability to successfully relieve pain, and a radical or ‘riskier’ option was more potent, with a lower probability to successfully relieve pain (Fig. 1A). The findings suggested that making a medical decision is associated with complicated assessment of risk, which relates to the unpredictability of an outcome^7^. However, these aspects of medical decision-making have not been systematically investigated.

Evidence from functional magnetic resonance imaging (MRI) studies has revealed that when an individual is assessing the gains and losses for a risky financial decision, the anterior insula (aINS) and the nucleus accumbens (NAc), as the core components of the risk-related network, were frequently activated^8^,^9^. The aINS activation is closely associated with anticipation of aversive stimulus^10^, and its functional connectivity with the dorsal anterior cingulate cortex (dACC) would reflect a heightened salience about pain^11^. The aINS activation may represent the degree of uncertainty of an outcome^12^ and play a critical role in the aversion of losses^13^,^14^. In contrast, the NAc activation is frequently reported in the scenario when an individual pursued gains^14^,^15^, echoing its role in the mesolimbic dopaminergic system^16^. Activation of the mesolimbic system is associated with pain relief, a desirable status that can be considered as a reward^17^,^18^. The functional roles of the aINS and the NAc are parallel to the processing of pain and pleasure^17^,^19^, which are major motivators for medical care-seeking. Furthermore, the variation in intrinsic brain signatures, including resting-state (rs) functional connectivity (FC) and grey matter volume (GMV), is associated with the individual differences in risk-taking tendency^20^,^21^,^22^. The findings imply that the variation in intrinsic brain signatures, of the aINS and the NAc may account for the individual differences in risk-taking tendency.

We here adopted the ADT for assessing the risk-taking tendency regarding the choice of analgesic treatment, which was quantified as the risk preference index (RPI). We analyzed the structural (GMV) and functional (rs-FC connectome) signatures of a risk-related network composed of 26 brain regions. We hypothesized that at the aINS and the NAc, network degree centrality (DC), FC and GMV, would be correlated with the individual differences in RPI.

## Methods

### Participants

The current observational study adopted a cross-sectional design. Thirty-six participants (18 females) between ages of 21 and 46 years (M = 28.1; SD = 5.3) were recruited in at the university campus (see Table 1 for the demographic and clinical profiles of the participants). The sample size was decided based on power analysis, using G*Power 3.1.9.2^23^, for a two-tailed bivariate correlation analysis with alpha = 0.05, power = 0.8, and an medium effect size 0.45. All the participants were recruited via posted advertisement. None of the participants had reported a history of chronic pain or had been previously diagnosed with a psychiatric disorder (see Table 1 for the detailed demographic and behavioral results).

### Research Ethics

The study protocol and the relevant methods were approved by the Institutional Review Board of Taipei Veterans General Hospital (IRB code: 2013–080021BCY). All methods were performed in accordance with the relevant guidelines and regulations. All of the participants provided written informed consent before participating in this study.

### Assessment of Prior Pain Experience and Imagined Pain Relief

Previous studies have shown that one’s prior experience of clinical pain and subjective satisfaction (i.e., utility) of pain reduction may play a key role in the choice of analgesic treatment^24^,^25^. We first assessed the participants’ prior experience of clinical pain, including toothache, headache and stomach ache, and their imagined pain relief in three conditions: from 9 to 0 (Δ9 → 0), 9 to 3 (Δ9 → 3) and 9 to 6 (Δ9 → 6), based on an 11-point numerical rating scale.

Before performing the ADT, the participants were asked to rate their most intense prior experience of clinical pain, respectively for toothaches, headaches and stomach aches, using a 0–100 scale (0 = no pain, 100 = worst possible pain). Subsequently, we assessed the utility (i.e., subjective satisfaction ref. 26) of pain reduction, which was quantified as imagined pain relief^5^, the degree of satisfaction about pain reduction. The participants were asked to imagine that they were experiencing an acute pain (toothache, headache and stomach ache), with an intensity of 9, based on an 11-point numerical rating scale (NRS) (0 = non-painful and 10 = extremely painful). Subsequently, they were asked to rate imagined pain relief for the following figurative conditions of pain reduction (1) from 9 to 0 (ΔP9 → 0), (2) from 9 to 6 (ΔP9 → 6), and (3) from 9 to 3 (ΔP9 → 3). Imagined pain relief was rated based on a 0–100 numerical scale (0 = no relief and 100 = the strongest relief).

### Analgesic Decision-making Task

We assessed the participants’ preferences of analgesic treatment in 22 figurative scenarios where they imagined that they were in pain^5^,^6^. The task is implemented as a pencil-and-paper questionnaire that consisted of three sub-tasks: the ‘Analgesic Effect (ANE) task (8 scenarios), the ‘Adverse Effect’ (ADE) task (8 scenarios), and the ‘Time-course Effect’ (TE) task (6 scenarios), presented in a counterbalanced order. The participants needed to imagine that they were experiencing pain at 9, based on the same NRS used in the assessment of prior pain experience, and they would make a choice between two figurative analgesic treatments to reduce the pain. The design of each sub-task was based on our previous studies^5^,^6^: (i) in the ANE task, the riskier (radical) treatment was always more potent but less likely to work successfully, and the riskless (conservative) treatment always was less potent but more likely to work successfully. (ii) In the ADE task, the riskier treatment was always more potent but more likely to induce an adverse effect, and the riskless treatment was less potent but less likely to induce an adverse effect. (iii) In the TE task, the riskier treatment was always more potent over the long run but was slower to reduce pain, and the riskless treatment was less potent over the long run but was quicker to reduce pain. The potency of pain reduction and other attributes (i.e., the probabilities that a treatment will work and an adverse effect will occur, and the time delayed for maximal effect) were parametrized across the 22 scenarios. The participants were asked to weigh the potency of pain reduction and the other attributes, when making their decisions. Detailed parameters of the experimental design are illustrated in Supporting Information and our previous studies^5^,^6^.

### Quantification of Individual Risk-taking Tendency

We quantified the tendency of risk-taking as the frequency to choose the ‘riskier’ option in the ADT. For all the 22 scenarios (8 for the ANE task, 8 for the ADE task and 6 for the TE task), we calculated the Risk Preference Index (RPI) as follows:


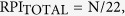


N is the frequency that a participant chose the riskier option. A higher RPI indicated a stronger tendency that the participant would choose the riskier treatment, i.e., the treatment that has a stronger pain-relieving effect, regardless of the influence of probability, adverse effect or time-course of therapeutic effect. To further investigate risk-taking tendency in each sub-task, we analyzed the frequency that a participant chose the riskier option in the ANE task (N_ANE_), the ADE task (N_ADE_) and the TE task (N_TE_). The RPI for each task was calculated as a proportion (RPI_ANE_ = N_ANE_/8, RPI_ADE_ = N_ADE_/8, and RPI_TE_ = N_TE_/6).

### Acquisition and Pre-processing of Imaging Data

Resting-state functional MRI (rs-fMRI) and T1-weighted magnetic resonance imaging (T1-MRI) were performed at the 3 T MRI Laboratory of National Yang-Ming University, using a 3 Tesla Siemens MRI scanner (Siemens Magnetom Tim Trio, Erlangen, Germany). The rs-fMRI images were acquired with the following parameters: gradient echo planar imaging (EPI) with T2* weighted sequence ([TR] = 2000 ms, [TE] = 20 ms, matrix size = 64 × 64 × 40, voxel size = 3.4 × 3.4 × 3.4 mm^3^, and 183 volumes in total). The T1-MRI images were acquired with the following parameters: a high-resolution sequence ([TR] = 2530 ms, [TE] = 3.02 ms, matrix size = 256 × 256 × 192, voxel size = 1 × 1 × 1 mm^3^). During rs-fMRI, the participants were instructed to be relaxed, remain awake, and keep their eyes open and fix on a cross symbol on the screen.

Pre-processing of the rs-fMRI data was performed using the Data Processing Assistant for Resting-State fMRI^27^ and the Resting-State fMRI Data Analysis Toolkit^28^. The first three scans were discarded for magnetic saturation effects. The imaging data were slice-timing corrected, realigned for head motion, normalized to the Montreal Neurological Institute (MNI) template, and smoothed with an 8 mm Gaussian kernel. The time series was de-trended and band-pass filtered (0.01–0.08 Hz) to extract the low-frequency oscillating components contributing to intrinsic functional connectivity. A regression model was used to remove the spurious or non-specific effects from the following covariates: (a) the parameters of translational and rotational motion, obtained from realignment, (b) the mean signal within the lateral ventricles, and (c) the mean signal within the deep white matter. The regression of whole-brain global signal was not performed, due to the debate on its effect^29^.

### Analysis of Behavioral Data

The difference in prior experience of clinical pain (i.e., toothache, headache and stomach ache), the difference in imagined pain relief (i.e., ΔP9 → 0, ΔP9 → 3 and ΔP9 → 6), and the difference in RPI in all three sub-tasks (i.e., ANE, ADE and TE), were respectively investigated, using the Friedman test. Post-hoc pairwise comparisons were performed using the Wilcoxon-signed rank test, with Bonferroni correction for multiple comparison. We adopted the non-parametric methods due to the non-normality of data (Kolmogorov-Smirnov test, P < 0.1, see Table 1). We first compared the pain ratings from the three categories of clinical pain. A statistically significant difference of the pain ratings would suggest that some of the clinical pain may have a stronger impact on one’s prior pain experience, compared to the others. Based on our previous findings^5^,^6^, we expected that the imagined pain relief would be significantly higher in ΔP9 → 3, compared to ΔP9 → 6. To characterize the association between imagined pain relief and the RPIs, we performed partial correlation analyses between the variables, controlled for sex and age. Based on our previous findings^6^, we expected that imagined pain relief from 9 to 6 (i.e., Δ9 → 6), which represents the individual utility of pain reduction, was correlated with the RPI.

### Analysis of Imaging Data

Acquisition and pre-processing of the imaging data were documented in SI Methods. Our general hypothesis focused on the association between risk-taking tendency and functional/structural signatures of the aINS and the NAc. To test the hypothesis, we performed the following three correlation-based analyses:

- On the pattern of rsFC connectome, we performed partial correlation analyses to investigate the association between DC of the bilateral aINS and NAc and RPI, controlled for sex and age. The analyses were performed, respectively for RPI_TOTAL_, RPI_ANE_, RPI_ADE_ and RPI_TE_. We hypothesized that degree centrality (DC) of the aINS (DC_aINS_L_/DC_aINS_R_) and the NAc (DC_NAc_L_/DC_NAc_R_) is associated with the individual RPI. To test the hypothesis, we constructed a risk-related network, primarily based on imaging meta-analysis (see Fig. 2A, also see Supporting Information for detailed procedures). The network consisted of 26 bilateral brain regions (i.e., network nodes) (see Fig. 2B for the regions, and also Table 2 for their definition). A higher DC indicates that a node is more connected with the other nodes, i.e., playing an important role in the network.
- On the aINS/NAc functional connectivity, to understand the pattern of their connections with the other brain regions, we further performed exploratory whole-brain seed-based FC analyses, using the bilateral aINS and the NAc, respectively, as the seeds. We tested a multiple regression model that modeled participants’ sex, age as nuisance regressors and RPI (separately for RPI_TOTAL_, RPI_ANE_, RPI_ADE_, and RPI_TE_) as the predictors, and the seed-based functional connectivity as the dependent variable. The bilateral aINS and the NAc were respectively used as the seed. The analyses would reveal the brain cluster which connectivity was positively correlated with RPI. A cluster would be considered statistically significant with a threshold of intensity (uncorrected P [P_unc_] < 0.005) and a threshold of cluster size (familywise-error corrected P [P_FWE_] < 0.05).
- On the structural signature, we first performed a region-of-interest (ROI)-based analysis, focusing on the bilateral aINS and NAc. We tested a multiple regression model that modeled the sex, age, total brain volume, all as nuisance regressors, and RPI (separately for RPI_TOTAL_, RPI_ANE_, RPI_ADE_, and RPI_TE_) as the predictors, and grey matter volume (GMV) as the dependent variable. Because we focused on the role of the aINS and the NAc, we performed an ROI-based analysis^30^, using the masks of bilateral aINS and NAc as the ROIs (see Table 2 for ROI definition). A cluster would be considered statistically significant with a threshold of intensity (uncorrected P [P_unc_]<0.001) and cluster size (familywise-error corrected P [P_FWE_]<0.05, corrected for small volume).

### Analysis of the Mediating Effect of the aINS Connectome

Findings from the previous analyses revealed that RPI_TOTAL_ was positively correlated with both the functional signatures (i.e., DC_aINS_L_/DC_aINS_R_) and structural signatures (i.e., GMV_aINS_R_) (see Results). To further clarify the association between these variables, we performed a mediation analysis^31^ to investigate if the association between GMV_aINS_R_ and RPI was mediated by DC_aINS_L_/DC_aINS_R_ (i.e., the mediator variable). We assigned GMV as the independent variable, RPI as the dependent variable and DC as the mediator. DC of the right and the left aINS was used, separately, as the mediator. Sobel test was performed to examine the significance of the ‘indirect model’, which represents that a mediational effect of DC.

### Predicting Risk-taking Preference from Behavioral and Brain Signatures

The abovementioned results pointed to the conclusion that functional connectome of the left aINS plays a key role in the individual differences of risk-taking tendency. In addition, imagined pain relief and prior pain experience may also guide one’s analgesic decisions. Therefore, we performed a multiple regression analysis for testing the ad hoc hypothesis that functional connectome of the aINS would predict one’s risk-taking tendency in medical decision-making. We modeled RPI_TOTAL_, as the dependent variable, and the following variables as the predictors: (a) imagined pain relief ΔP9 → 6, (b) prior pain experience, and (c) DC_aINS_L_, which was a significant mediator of RPI, according to the results from the mediational analysis (see Results). The variables ΔP9 → 6 and prior pain experience were log-transformed for normality. We first investigated the covariation between the predictors. The three predictors were not significantly correlated with each other. Secondly, we performed a regression analysis, using the input model, to investigate the predictors which predicted RPI_TOTAL_ with a statistically significance. Thirdly, we performed an analysis, using the stepwise method, to investigate the relative effect of prediction from the behavioral and rsFC-connectome predictors.

## Results

### Behavioral Findings

**Figure 1:**
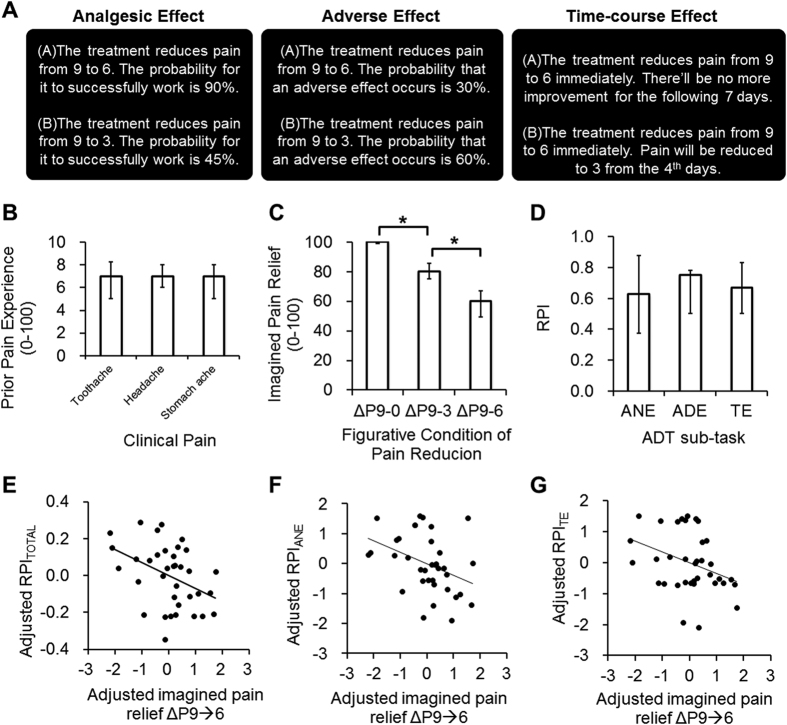
The Analgesic Decision-making Task (ADT). In the Analgesic Effect sub-task, the participant needs to choose between the two treatment options, which are opposite in pain-relieving potency and the probability that the treatment would successfully work. In the Adverse Effect sub-task, the participant needs to choose between the options that are opposite in pain-relieving potency and the probability to have an adverse effect. In the Time-course Effect sub-task, the participant needs to choose between the options that are opposite in pain-relieving potency and the time for the treatment to reach its maximal effect (Panel A). The difference in pain experience across the three categories of clinical pain was statistically insignificant (Friedman test, χ^2^_(2)_ = 0.55, P = 0.76) (Panel B). The difference in imagined pain relief across the three figurative conditions was statistically significant (Friedman test, χ^2^_(2)_ = 71.51, P < 0.001). The subsequent pairwise comparison showed that the imagined pain relief in Δ9 → 0 was significantly higher than those in Δ9 → 3 (P < 0.01), and the imagined pain relief in Δ9 → 3 was significantly higher than those in Δ9 → 6 (P < 0.01) (Panel C). The difference in RPI across the three sub-tasks of the ADT was statistically insignificant (Friedman test, χ^2^_(2)_ = 4.46, P = 0.11) (Panel D). In Panel B–D, the bar denotes the median and the horizontal lines denote the first and the third quartiles. The correlation was statistically significant between imagined pain relief Δ9 → 6 and the RPI calculated from all task scenarios (RPI_TOTAL_) (Panel E), RPI_ANE_ (Panel F) and RPI_TE_ (Panel G).

**Figure 2:**
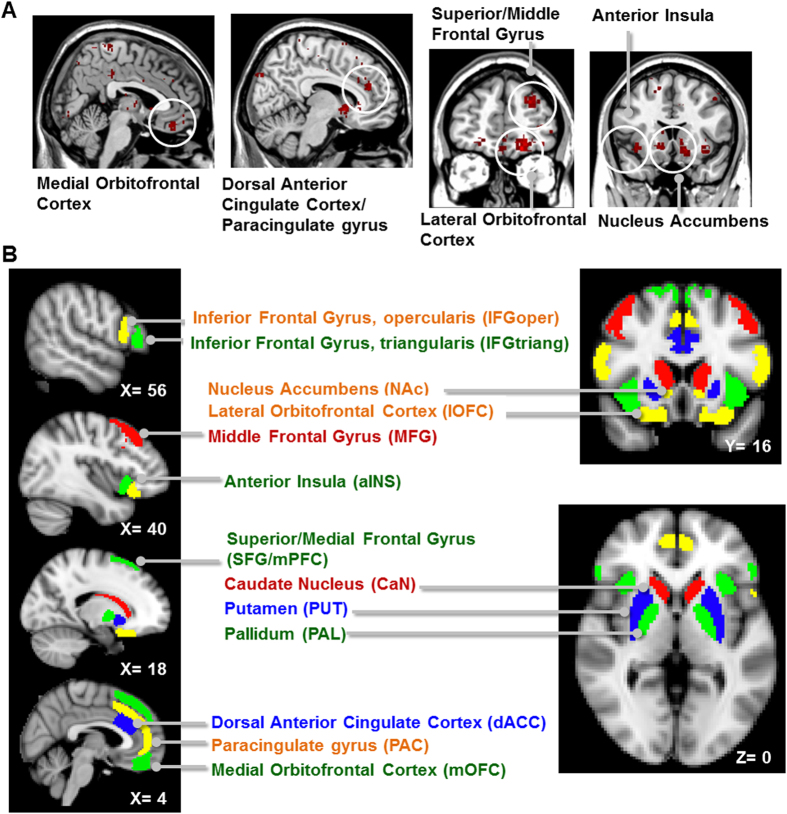
The risk-related network derived from an imaging meta-analysis implemented using Neurosynth (see SI Methods for detailed procedures). The network composed of anterior insula, nucleus accumbens, the orbitofrontal cortex, the anterior cingulate gyrus, and the lateral and medial prefrontal cortex (Panel A). The brain regions (i.e., the nodes) of the risk-related network in the rsFC connectome analysis (Panel B).

**Figure 3:**
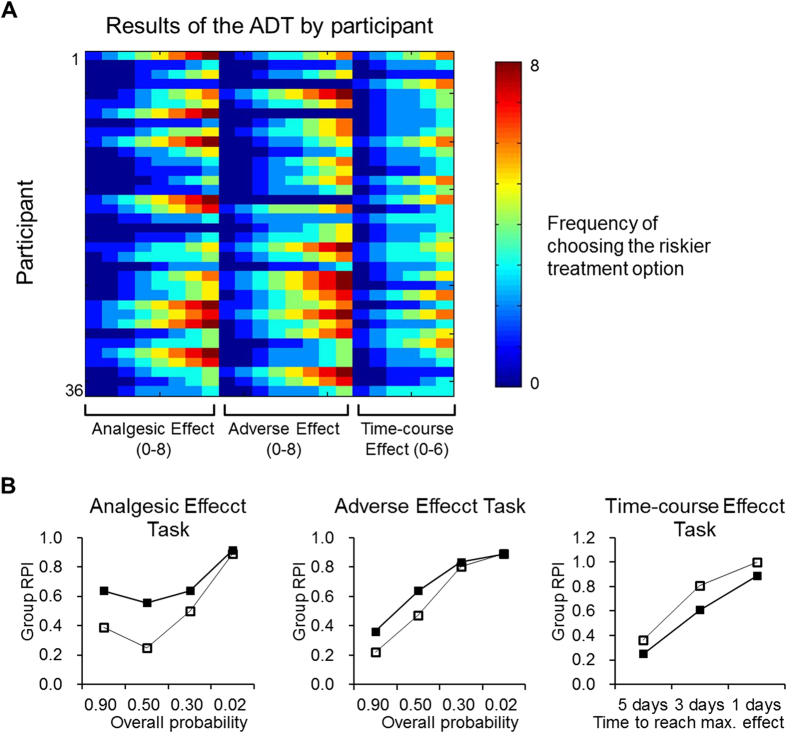
Results of the Analgesic Decision-making Task. The color image presents the frequency that a participant would choose the riskier treatment option. The value ranges from 0–8 in the Analgesic Effect Task (ANE) and the Adverse Effect task (ADE), which both consisted of 8 scenarios. The value ranges from 0–6 in the Time-course Effect Task (TE), which consists of 6 scenarios (Panel A). The change of risk-taking preference in different conditions of the sub-tasks. In the ANE task, the risk-taking preference of the group (i.e., group RPI) increases as the overall probability to have an analgesic effect decreases. In the ADE task, group RPI increases as the overall probability to have an adverse effect decreases. In the TE task, group RPI increases as the time delayed to reach maximal effect decreases (Panel B).

Prior clinical pain did not significantly differ between the three categories of clinic pain (Fig. 1B), suggesting a homogeneous experience across different clinical pain. Imagined pain relief increased across the three conditions (Δ9 → 6 < Δ9 → 3 < Δ9 → 0, Fig. 1C). The findings indicated that the participants were sensitive to the changes in the utility of pain reduction^5^,^6^. Across the three ADT sub-tasks – the Analgesic Effect task (ANE), the Adverse Effect task (ADE) and the Time-course Effect task (TE) – we did not find significant difference in the RPI (Fig. 1D). Further investigation revealed that the correlation was statistically significant between imagined pain relief Δ9 → 6 and the RPI calculated from all task scenarios (RPI_TOTAL_) (r = −0.41, P = 0.017, Fig. 1E), RPI_ANE_ (r = −0.38, P = 0.025, Fig. 1F) and RPI_TE_ (r = −0.35, P = 0.042, Fig. 1G), but not RPI_ADE_ (r = −0.07, P = 0.7). The results confirmed our previous findings that risk-taking tendency is associated with subjective utility of pain reduction (see Fig. 3 and SI Methods for detailed results from the ADT).

### Analysis of rsFC Connectome

**Figure 4:**
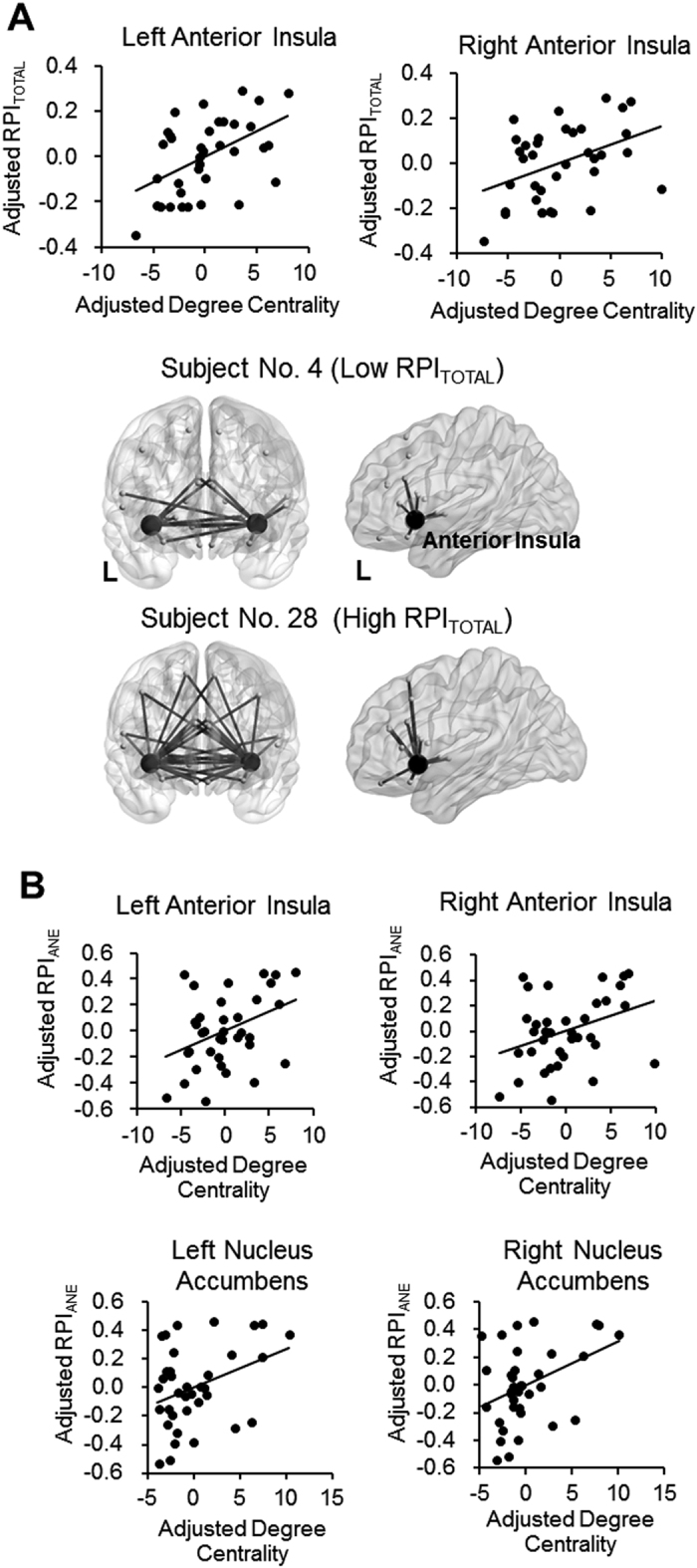
Results of the rsFC connectome analysis. DC of the bilateral aINS is positively correlated with RPI_TOTAL_, controlled for participants’ sex and age. The lower panel revealed that a participant with a higher RPI_TOTAL_ showed denser FC at the bilateral aINS, compared to a participant with a lower RPI_TOTAL_ (Panel A). DC of the bilateral aINS and the NAc is positively correlated with RPI_ANE_, but not RPI_ADE_ or RPI_TE_, controlled for participants’ sex and age (Panel B).

In general, partial correlation analyses revealed that RPI_TOTAL_ was positively correlated with both DC_aINS_L_ (r = 0.49, P = 0.002) and DC_aINS_R_ (r = 0.41, P = 0.014), controlled for participants’ sex and age (Fig. 4A). In terms of each sub-task, RPI_ANE_ was positively correlated with bilateral DC_aINS_ and bilateral DC_NAc_ (Fig. 4B), confirming our hypothesis. In contrast, RPI_ADE_ and RPI_TE_ did not show significant correlation with DC_aINS_ or DC_NAc_.

### Analysis of aINS/NAc Functional Connectivity

**Figure 5:**
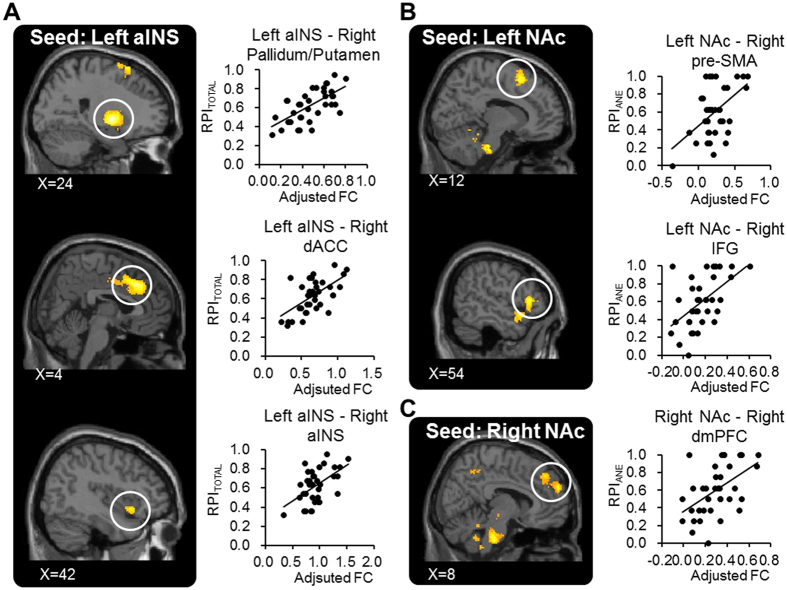
Results of the analyses of aINS/NAc FC. RPI_TOTAL_ is positively correlated with the rsFC between left aINS and multiple brain regions including the right pallidum and putamen, the right dACC and the right aINS (Panel A). RPI_ANE_ is positively correlated with the rsFC between the left NAc and the right pre-SMA/IFG (Panel B). RPI_ANE_ is positively correlated with the rsFC between the right Nac and the right dmPFC (Panel C). In all panels, the clusters larger than 350 voxels were visualized.

We found that the individual RPI was significantly positively correlated with the FC between the bilateral aINS/NAc and multiple brain regions (Table 3). Notably, RPI_TOTAL_ was positively correlated with the FC between the left aINS and the bilateral dorsal anterior cingulate cortex (dACC)/the right pallidum and putamen/the right aINS (Fig. 5A) and the FC between the right NAc and the right paracingulate cortex (PAC)/frontal pole. RPI_ANE_ was positively correlated with the FC between the left aINS and the bilateral PAC/dACC, the FC between the left NAc and the pre-Supplementary Motor Area (pre-SMA)/the right inferior frontal gyrus (IFG) (Fig. 5B), and the FC between the right NAc and the right dmPFC (Fig. 5C). RPI_TE_ was positively correlated with the FC between the left aINS and the dorsomedial prefrontal cortex (dmPFC)/the right IFG/premotor cortex.

### Analysis of Grey Matter Volume

**Figure 6:**
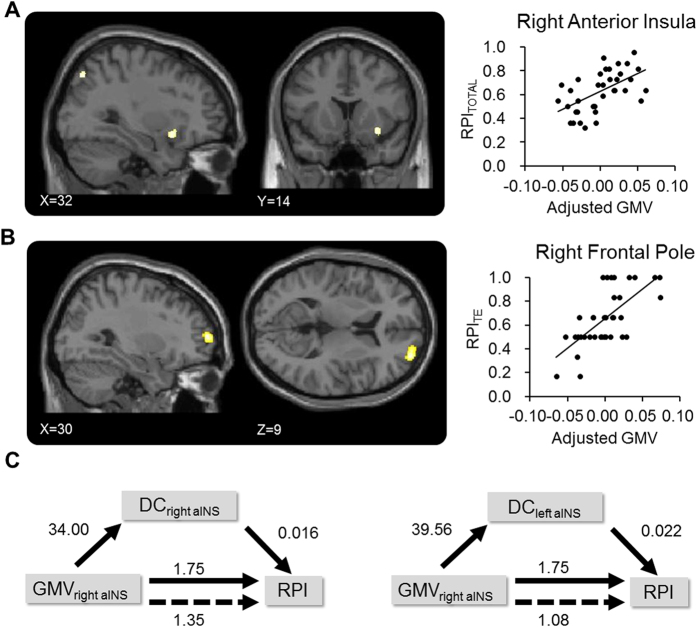
Results of the structural signatures and mediation analysis. GMV of the right aINS is positively correlated with RPI_TOTAL_ (Panel A). GMV of the right frontal pole is positively correlated with RPI_TE_ (Panel B). The mediation analysis revealed that the association between right aINS GMV and RPI_TOTAL_ is mediated by DC of the left aINS. The number denotes the non-standardized coefficient from the regression model that predicts the relationship between the IV, DV and mediator. All models are controlled for sex and age (Panel C).

We found that RPI_TOTAL_ was positively correlated with the GMV at the right aINS (GMV_aINS_R_) ([x,y,z] = [32,14,−12], Z = 3.64, cluster-based P_FWE_ = 0.011, corrected for small volume) (Fig. 6A). In contrast, RPI_ANE_, RPI_ADE_ and RPI_TE_ were not significantly correlated with either GMV_aINS_ or GMV_NAc_. Additionally, we performed a whole-brain exploratory analysis to investigate the brain region that showed revealed significantly positive correlation between the GMV and RPI_TOTAL_, RPI_ANE_, RPI_ADE_ and RPI_TE_. A stringent statistical threshold (family-wise error-corrected, P = 0.05) was adopted in the analysis. The only above-threshold cluster in the whole-brain analysis is the right prefrontal cortex, which GMV showed positive correlation with RPI_TE_ ([x,y,z] = [30,60,9], Z = 4.82, P_FWE_ = 0.027) (Fig. 6B).

### Analysis of the Mediating Effect of the aINS Connectome

We found that the association between GMV_aINS_R_ was significantly mediated by the DC_aINS_L_ (Sobel test, T = 0.21, P = 0.035) (Fig. 6C, also see Table 4 for detailed results). The findings suggested that the association between brain structure and RPI could be mediated by the functional connectome of the aINS.

### Predicting Risk-taking Preference from Behavioral and Brain Signatures

We first investigated the covariation between the predictors. The three predictors were not significantly correlated with each other. Secondly, we performed a regression analysis using the input model. The result showed that all the three predictors explained 38.5% of the variation of RPI_TOTAL_ (adjusted R^2^ = 0.385, P = 0.001). The significant predictors were DC of the left aINS (beta = 0.471, P = 0.001) and imagined pain relief (beta = −0.398, P = 0.007). In contrast, prior experience of pain was not a significant predictor. Thirdly, we performed an analysis using the stepwise method. The results showed that DC of the left aINS was the predominant predictor, which explained 26.1% of the variation of RPI_TOTAL_ (ΔR = 0.26, P = 0.001). Additionally, imagined pain relief explained 12% of the variation (ΔR = 0.12, P = 0.016).

## Discussion

### Summary of the Major Findings

In a medical context, decision-making is associated complicated assessment of gains, losses and uncertainty of outcomes^5^,^6^. Previous studies mostly focused on the behavioral and brain mechanisms underlying financial decision-making^14^,^32^. We here provide novel evidence about the brain mechanisms underlying decision-making in a medical context. Confirming our hypothesis, we found the aINS, a ‘core component’ of the risk-related network^9^ is associated with the individual differences in risk-taking tendency, assessed by the ADT. Specifically, we found that, on rs-FC connectome, the higher DC of the bilateral aINS and NAc, the stronger tendency or higher frequency (i.e., a higher RPI) that the participants would choose a riskier treatment to relieve pain (Fig. 4). FC between the aINS, the NAc and multiple brain regions, predominantly the SFG, the dACC and the medial frontal cortex, was positively correlated with RPI (Fig. 5). On structural signatures, we found that the RPI was positively correlated with the GMV at the right aINS (Fig. 6) and such an association was mediated by DC_aINS_L_. Finally, the regression model revealed that both the functional connectome (i.e., DC_aINS_L_) and psychological utility (i.e., imagined pain relief) can predict risk-taking tendency. Altogether, the findings suggested that the functional and structural brain signatures of the aINS are associated with the individual differences of risk-taking tendency in the context of medical decision-making.

### Neural Correlates of Risk-tendency of Analgesic Decision-making

Our findings fit into the proposal that during financial decision-making, risk assessment is associated with the activation of a ‘risk matrix’^9^,^33^. In such a network, the aINS plays a key role during the anticipatory phase of decision-making, when the participants need to assess the risk related to outcomes. Consistently, we found a significant role about the aINS in medical decision-making. The aINS is associated with not just the anticipation of incoming painful stimuli^11^, but also the emotional experience of recalled/imagined pain^34^. Therefore, a denser connection of the aINS (i.e., a higher DC_aINS_) during the resting status may reflect a heightened salience about the current status, which would direct the individual towards future pain-related experience. Consistently, the seed-based FC analysis showed that the aINS-dACC connectivity, i.e., the main component of the salience network^35^, and the connectivity between the right and the left aINS, were correlated with RPI_TOTAL_ (Fig. 5A). Such an increased saliency about pain would motivate the individual to take a riskier option, which would deliver a stronger pain-relieving effect. Consistent with this view, we found that functional connectome and FC of the NAc was associated with RPI, especially in the ANE task, the sub-task that required a participant to consider the probability to have their pain relieved (Figs 4B and 5B). In accordance with our results, evidence from financial decision-making tasks has revealed that the NAc activation was associated with the anticipation preceding the selection of a risky option, which would receive more gains, compared to a riskless option^14^,^15^. Notably, the patients with chronic pain, compared to healthy controls, showed a higher impulsivity to gain in a gambling task, and this impulsivity was associated with the changes in NAc connectivity^36^. As part of the mesolimbic dopaminergic system, the NAc plays a key role in processing of the pleasure from a reward^17^. Altogether, the findings suggested that a stronger tendency of choosing the riskier analgesic option may be associated with a higher salience about pain and a greater expected reward for pain relief.

We noted that the findings were not all consistent through the three sub-tasks. In the ADE tasks, neither DC_aINS_ nor DC_NAc_ was associated with RPI. Consistently, FC of these regions was not significantly correlated with RPI (Table 3). The contrasting findings from the ANE and the ADE tasks may suggest distinct the behavioral and brain mechanisms underlying the risk decision-making for gains (i.e., pursing analgesic effect) and losses (i.e., avoiding adverse effect). In the ANE task, the decision is oriented to pursue a reward, and the NAc would play a dominant role. The RPI was positively correlated with FC between the bilateral NAc and the brainstem as well as the hippocampus (Table 3). The NAc-hippocampus connectivity may reflect the effect of motivational significance on memory formation, bridging past experience with future decisions^37^. In contrast, in the ADE task, the decision was oriented to avoid harm, rather than to pursue a reward. Notably, our behavioral results showed that the behavioral index RPI_ANE_, but not RPI_ADE_, is associated with imagined pain relief (Fig. 1). The behavioral patterns were consistent with the imaging findings, showing a distinct mechanism underlying medical decisions of different orientations. Finally, in the TE task, we noted that FC between the left aINS and multiple regions in the dorsolateral prefrontal cortex (DLPFC), including the SFG, the MFG and the premotor cortex, was positively correlated with the RPI (Table 3). In the delayed-discounting task, the DLPFC regions composed of the time network, which reflected the sensitivity to time delay^21^. The DLPFC is associated with deliberation and planning^38^, and plays a key role in inhibiting an impulsive decision, i.e., seeking a quick but smaller reward^39^. In our TE task, increased FC of these regions may reflect the tendency that a participant would deliberate the future benefits of pain relief (i.e., preferring a riskier option) and inhibit the impulsivity for a quick but lesser relief.

### Limitations of the Study and Further Considerations

Our findings need to be interpreted based on the following limitations of study design. First, the ADT paradigm is designed based on the hypothetical choice paradigm, which has been widely used in assessing individual preference of monetary decision-making^32^. Such a paradigm would be, ecologically, more consistent with the scenario of making medical decisions, i.e., the situation when a patient needs to make a decision before she/he actually perceives the treatment effect. It should be noted that the results from a hypothetical choice paradigm can be different from an experience-based paradigm^40^. In the latter paradigm, decision makers receive feedback, such as the pain-relieving effect, from their decisions. Secondly, though the ADT was specifically designed to simulate a clinical pain-relieving scenario, it was simplified in many aspects. For example, we did not investigate the influence of financial costs about the treatment and assumed it is the same across all the task scenarios. The simplification may compromise the ecological validity of the ADT. Thirdly, the participants included in the study all received a higher degree of education. The homogeneity in education level suggested that the participants were able to understand the decision-making scenarios, which require basic literacy and numeracy. Our conclusion may not be generalized to the illiterate or innumerate people. Finally, we aimed to investigate the association between functional/structural signatures and individual differences in risk-taking. Such a correlation-based observation cannot elucidate the causal relationship between brain signatures and behavioral variations. Our findings may imply that a greater GMV and stronger functional connection at the aINS would predispose the tendency of risk-taking in making medical decisions. Conversely, it may imply that a long-term experience about making riskier decisions may, in a long run, reshape the functional and structural signatures, as an effect of brain plasticity.

### Clinical Implications

The current findings revealed that both structural and functional signatures of the aINS would reflect one’s risk-taking tendency. The clinical significance of this finding can be related to the role of the aINS in psychiatric disorders and chronic pain. Heighted activation of the aINS was associated with increased salience and perceived threat to pain^11^,^41^. It is noteworthy that the aINS plays a key role in anticipating aversive stimuli (ref. 11, especially in the anxiety-prone individuals^10^ Alterations in brain processing of anticipatory stimuli can be associated with the development of psychiatric disorders, such as generalized anxiety^42^. Consistently, in our study, the ADT requires the participants to anticipate experience of pain relief during decision-making. Therefore, changes in the aINS signatures implied that the individual suffer from psychiatric disorders or chronic pain may show a different risk-taking behavior, compared to healthy controls. The association between individual traits, their medical history and risk-taking tendency, would be clinically significant issue and require further investigation.

## Conclusions

Our novel behavioral and neuroimaging evidence suggests that the functional and structural brain signatures of the aINS are associated with the individual differences of risk-taking tendency in the context of analgesic decision-making.

## Additional Information

**How to cite this article**: Lin, C.-S. *et al*. Functional and Structural Signatures of the Anterior Insula are associated with Risk-taking Tendency of Analgesic Decision-making. *Sci. Rep.* **6**, 37816; doi: 10.1038/srep37816 (2016).

**Publisher's note:** Springer Nature remains neutral with regard to jurisdictional claims in published maps and institutional affiliations.

## Supplementary Material

Supplementary Information

## Figures and Tables

**Table 1:** Demographic and Behavioral Data.

		N	Mean	SD	Max	Min	Normality (*P*)^a^
**Demographic**							
Gender	Female	18					
	Male	18					
Age	Total		28.1	5.3	46	21	
	Female		28.2	6.5	46	21	
	Male		27.9	4.0	38	23	
Education	University/Postgraduate Degree	36					
**Behavioral**							
Prior Pain Experience							
Toothache			6.4	2.8	10	0	
Headache			6.8	1.9	10	2	
Stomach ache			6.2	2.6	10	0	
Imagined Pain Relief							
ΔP9 → 0			98.6	2.9	100	90	
ΔP9 → 3			79.9	8.8	100	60	
ΔP9 → 6			57.9	16.5	87	20	
Risk Preference Index (RPI)							
RPI_TOTAL_			0.63	0.17	1.0	0.3	0.20
RPI_ANE_			0.60	0.28	1.0	0	0.18
RPI_ADE_			0.64	0.28	1.0	0	
RPI_TE_			0.65	0.24	1.0	0.2	
Fear of Pain			28.5	4.6	36	18	0.18
Pain Catastrophizing			21.6	9.0	38	5	0.20
Trait Anxiety			45.4	5.8	56	28	0.20

N, number of participants; SD, standard deviation.

^a^Data was considered normally distribution based on the Kolmogorov-Smirnov test, *P *> 0.1. For the assessment of Fear of Pain, Pain Catastrophizing and Trait Anxiety, see Supporting Information.

**Table 2:** Selection of the Nodes and Definition of Brain Regions.

Brain region	Label	Side	Source^*^	
Superior Frontal Gyrus/Medial Frontal Gyrus	SFG	L	Harvard-Oxford Cortical Anatomy Atlas	#3
		R		#3
Middle Frontal Gyrus	MFG	L		#4
		R		#4
Inferior Frontal Gyrus, triangularis	IFGtriang	L		#5
		R		#5
Inferior Frontal Gyrus, opercularis	IFGoper	L		#6
		R		#6
Paracingulate Gyrus/Medial Frontal Gyrus	PAC	L		#28
		R		#28
Medial Orbitofrontal Cortex/Ventromedial Prefrontal Cortex	mOFC	L		#25
		R		#25
Lateral Orbitofrontal Cortex	lOFC	L		#33
		R		#33
Anterior Insula	aINS	L	Manual definition, based on^43^	
		R		
Dorsal Anterior Cingulate Cortex	dACC	L		
		R		
Caudate Nucleus	CaN	L	Harvard-Oxford Sub-cortical Anatomy Atlas	#5
		R		#16
Putamen	PUT	L		#6
		R		#17
Pallidum	PAL	L		#7
		R		#18
Nucleus Accumbens	NAc	L		#11
		R		#21

**Table 3:** Results of Seed-based Functional Connectivity Analyses.

(A) Positive correlation with RPI_TOTAL_																
Seed	Cluster within the decision-related network								Cluster outside the decision-related network							
	Label^1^		size	pFWE	Z	x	y	z	Label		size	pFWE	Z	x	y	z
Left aINS	Pallidum/Putamen	R	776	0.002	4.5	24	−2	0								
	aINS	R			3.4	42	16	−8								
	aINS/Frontal Orbital Cortex	R				30	8	−14								
	PAC/dACC	R	1443	<0.001	4.2	4	26	38								
	PAC/dACC	R			4.2	8	18	42								
	PAC/dACC	L			3.8	−6	24	34								
Right aINS	n.s.															
Left NAc	n.s.															
Right NAc	PAC/dACC	R	456	0.040	3.3	16	42	18								
	dmPFC	R			3.3	8	58	26								
	dmPFC	R			3.0	14	50	24								
**(B) Positive correlation with RPI**_**ANE**_																
	**Cluster within the decision-related network**								**Cluster outside the decision-related network**							
**Seed**	**Label**		**size**	**pFWE**	**Z**	**x**	**y**	**z**	**Label**		**size**	**pFWE**	**Z**	**x**	**y**	**z**
Left aINS	PAC/dACC	L	465	0.039	3.5	−4	12	44								
	PAC/dmPFC	L			3.3	−6	32	40								
	PAC/dACC	R			3.3	4	20	40								
Right aINS	n.s.															
Left NAc	dACC/PAC	L	575	0.013	3.8	−12	14	32	Parahippocamus	R	546	0.017	4.4	16	0	−36
	pre-SMA	R			3.7	12	14	56	Parahippocamus	R			3.6	26	−8	−36
	pre-SMA	R			3.5	6	18	60	Parahippocamus	R			3.5	20	−6	−44
	IFG, triangularis	R	595	0.011	3.5	54	26	8	Occipital Lobe	R	1122	<0.001	4.2	26	−100	12
	Frontal Orbital Cortex	R			3.4	46	20	−14	Occipital Lobe	R			3.6	40	−86	0
	Temporal Pole	R			3.4	56	12	−12	Occipital Lobe	R			3.4	28	−86	10
									Brainstem	L	485	0.030	4.2	−2	−18	−34
									Brainstem	R			3.7	2	−24	−38
									Brainstem	R			3.6	10	−24	−34
									Hippocampus	R	579	0.012	3.8	22	−32	−16
									Cerebellum, anterior lobe	R			3.7	16	−42	−18
									Hippocampus	R			3.6	26	−28	−8
Right NAc	dmPFC	R	665	0.005	3.7	8	58	26	Brainstem	L	2071	<0.001	4.7	−2	−20	−42
	dmPFC	R			3.4	10	40	44	Hippocampus	R			4.6	22	−32	−16
	dmPFC	L			3.2	−2	50	32	Hippocampus	R			4.4	26	−28	−8
									Parahippocamus	R	1269	<0.001	4.4	18	0	−38
									IGF, opercularis	R			3.5	52	12	−22
									Amygdala	L	496	0.023	3.7	−28	−2	−26
									Parahippocamus	L			3.7	−18	2	−38
									aINS	L			3.5	−38	6	−14
									Precuneus/SPL	L	426	0.048	3.4	−6	−56	48
									SPL	L			3.0	−8	−34	46
**(C) Positive correlation with RPI**_**ADE**_																
Left aINS	n.s.															
Right aINS	n.s.															
Left NAc	n.s.															
Right NAc	n.s.															
**(D) Positive correlation with RPI_TE_**																
Left aINS	IFG, opercularis	R	958	<0.001	4.8	50	10	22	Occipital Lobe	L	3255	<0.001	4.2	−28	−70	24
	MFG	R			3.8	46	22	24	SPL	L			4.0	−24	−48	40
	SFG	R	1447	<0.001	4.4	26	10	60	Occipital Lobe	L			3.9	−24	−64	28
	PAC/Premotor Cortex	R			4.2	10	14	46	Occipital Lobe	R	2586	<0.001	4.0	32	−66	34
	MFG	R			3.9	30	0	52	Occipital Lobe	R			4.0	24	−56	42
									SPL	R			3.8	40	−44	54
									SFG/Premotor Cortex	L	748	0.003	3.9	−22	10	52
									SFG/Premotor Cortex	L			3.7	−24	0	58
									SFG/Premotor Cortex	L			3.5	−16	−4	62
Right aINS									Occipital Lobe	L	587	0.016	3.6	−26	−68	26
									Occipital Lobe	L			3.5	−26	−96	22
									Cuneus	L			3.4	−20	−76	26
Left NAc	n.s.															
Right NAc	n.s.															

n.s., not significant.

^1^All the labels were surveyed according to the Harvard-Oxford Cortical/Subcortical Structural Atlas, based on FSLView v.3.2.0 (http://fsl.fmrib.ox.ac.uk/fsl/fslview/).

**Table 4:** Mediation Analysis of the association between Degree Centrality (DC), Grey Matter Volume (GMV) and Risk-taking Preference (RPI).

Mediator variable (MV)	Model 1: GMV(IV)-RPI(DV) association mediated by MV, controlled for sex and age			Model 2: GMV(IV)-RPI(DV) association mediated by MV, controlled for sex and age		
	DC of left aINS			DC of right aINS		
Path	Coef	SE	p	Coef	SE	p
Regress DV against MV	0.022	0.007	0.003	0.016	0.007	0.017
Regress M against IV	39.56	14.13	0.009	34.000	16.600	0.049
Regress DV against IV	1.754	0.647	0.011	1.754	0.647	0.011
Regress DV against IV and MV	(IV)1.081	0.681	0.123	(IV)1.350	0.666	0.051
	(MV)0.017	0.008	0.033	(MV)0.012	0.007	0.085
	Test statistic	p		Test statistic	p	
Sobel Test	2.1	0.035		1.5	0.113	

Coef: un-standardized coefficient.
