# Supplementary material for: Functional and Structural Signatures of the Anterior Insula are associated with Risk-taking Tendency of Analgesic Decision-making
Source: Sci Rep. 2016 Nov 28;6:37816. doi: 10.1038/srep37816 (PMC5124953; doi:10.1038/srep37816)
Supplement: Supplementary Information [file srep37816-s1.docx]

**Title**

Functional and Structural Signatures of the Anterior Insula are associated with Risk-taking Tendency of Analgesic Decision-making

**Running title**

Role of Anterior Insula in Analgesic Decision-making

**Authors**

Chia-Shu Lin^1^, Hsiao-Han Lin^1^, Shih-Yun Wu^1,2^

**Affiliations**

^1^Department of Dentistry, School of Dentistry, National Yang-Ming University

^2^Division of Family Dentistry, Department of Stomatology, Taipei Veterans General Hospital

**Corresponding author:**

Chia-shu Lin, DDS, MSc, PhD

Address: No. 155, Sec. 2, Linong Street, Taipei, 11221 Taiwan (ROC)

E-mail: winzlin@ym.edu.tw

Tel: +886-2-28267969

Fax: +886-2-28264053

Institutional URL: http://dod.web.ym.edu.tw/front/bin/home.phtml

**Supporting Information**

**Analgesic Decision-making Task (ADT).** The ADT is implemented as a pencil-and-paper questionnaire consisted of 22 figurative scenarios [^1^](#_ENREF_1)^,^[^2^](#_ENREF_2). The full ADT is divided into three sub-tasks: ‘*Analgesic Effect* (ANE) task (8 scenarios), the ‘*Adverse Effect*’ (ADE) task (8 scenarios), and the ‘*Time-course Effect*’ (TE) task (6 scenarios), presented in a counterbalanced order. The participants needed to imagine that they were experiencing pain at 9, based on an 11-point numerical scale (0 = not painful, 10 = extremely painful), and they would make a choice between two figurative analgesic treatments to reduce the pain. The design of each sub-tasks was based on our previous studies [^1^](#_ENREF_1)^,^[^2^](#_ENREF_2):

1. In the ANE task, the *riskier* (radical) treatment was always more potent but less likely to work successfully, and the *riskless* (conservative) treatment always was less potent but more likely to work successfully. The task consisted of 8 scenarios, which varied between the relative potency between the options (riskier vs. riskless), and the overall probability that the treatment would successfully work (riskier vs. riskless). The relative potency could be smaller (Δ9🡪3 vs. Δ9🡪6) or larger (Δ9🡪0 vs. Δ9🡪6). The overall probability varied from very high (45% vs. 90%), moderate (25% vs. 50%), low (15% vs. 30%) to very low (1% vs. 2%).
2. In the ADE task, the *riskier* treatment was always more potent but more likely to induce an adverse effect, and the *riskless* treatment was less potent but less likely to induce an adverse effect. The task consisted of 8 scenarios, which varied between the relative potency between the options (riskier vs. riskless), and the overall probability that an adverse effect would occur (riskier vs. riskless). The relative potency could be smaller (Δ9🡪3 vs. Δ9🡪6) or larger (Δ9🡪0 vs. Δ9🡪6). The overall probability varied from very high (90% vs. 45%), moderate (50% vs. 25%), low (30% vs. 15%) to very low (2% vs. 21%).
3. In the TE task, the *riskier* treatment was always more potent over the long run but was slower to reduce pain, and the *riskless* treatment was less potent over the long run but was quicker to reduce pain. The task consisted of 6 scenarios, which varied between the relative potency between the options (riskier vs. riskless), and the overall probability that the treatment would successfully work (riskier vs. riskless). The relative potency could be smaller (Δ9🡪0 vs. Δ9🡪3) or larger (Δ9🡪0 vs. Δ9🡪6). The time (days) delayed to reach maximal effect varied from 5 days, 3 days to 1 day.

Additionally, we assessed the pain-related personal traits, using the short-form Fear of Pain Questionnaire[^3^](#_ENREF_3)^,^[^4^](#_ENREF_4), the Pain Catastrophizing Scale[^5^](#_ENREF_5), and the Trait Anxiety scale from the State-Trait Anxiety Inventory[^6^](#_ENREF_6). As shown in Table 1, the scores are all normally distributed.

**Meta-analysis of the Risk-related Brain Regions.** Before constructing the risk-related network for the graph-based analysis of functional connectome, we selected the risk-related brain regions, primarily based on meta-analysis of imaging. The imaging meta-analysis was performed, using Neurosynth (http://neurosynth.org/), an Internet-based platform that performs automated synthesis of large-scale functional MRI data [^7^](#_ENREF_7). We search for the relevant imaging findings using the combination of keywords ‘risk & decision’, which resulted in 82 articles. The automatic synthesis created a ‘reverse inference’ map, which presents the pattern of brain activation preferentially related to decision-making under risk (Fig. 2*A*).

**Graph-based Network Analysis.** Our first hypothesis predicted that the medical decision about reward (i.e., pain relief) and the decision about loss (i.e., the adverse effect) would be associated, differentially, with the intrinsic brain signatures of the NAc and the aINS. To test the hypothesis, we performed a graph-based network analysis by constructing a network composed of the brain regions (i.e., the nodes) associated with decision-making under risk. Based on literature, the following 26 brain regions have been selected: (see Table II and Fig. 2*B* for the definition of the region-of-interest of the brain regions). Secondly, we defined the link (i.e., the edges) between each pair of node, according to the strength of association of their rs-fMRI time series. The weight of a link was quantified as the Pearson’s correlation coefficient of mean time series between two nodes. Therefore, for 26 nodes, an un-directional network with 325 edges was constructed. Finally, to quantify the importance of a node in the network, we calculated degree centrality (DC) of each node, which as defined as the sum of all weights between the node and the other nodes [^8^](#_ENREF_8). Therefore, a higher DC indicates that the node is more densely connected, as a ‘hub’ in the network.

**Results of the Analgesic Decision-making Task.** Descriptive, we first checked the individual variation in preference. We calculated the cumulative frequency that a participant chose the riskier option, separately for the Analgesic Effect Task (ANE), the Adverse Effect Task (ADE) and the Time-course Task (TE). As shown in Fig. 3*A*, we found pronounced individual differences in the pattern of preference: while some participants (e.g., #1) preferred the riskier options in all three sub-tasks, the others (e.g., #36) preferred the riskless options.

Quantitatively, we investigated the influence of (a) the overall probability for a treatment be effective, (b) the overall probability that an adverse effect would occur, and (c) the time delay before the treatment reaches its maximal effect, respectively, in the ANE, the ADE and the TE tasks. We calculated the index group RPI, which represents the averaged frequency to choose the riskier option across all participants, respectively for each scenario. Cochran test was performed to compare the group RPI from different scenarios. The results are summarized as follows:

1. For the ANE task, group RPI significantly differed between each condition of overall probability, when relative ΔP is smaller (Q_(3)_ = 39.5, P < 0.001) and when relative ΔP is larger (Q_(3)_ = 17.3, P = 0.001). Group RPI increased as the overall probability decreased (Fig. 3*B*, the left panel). The results confirmed our previous findings that the overall probability for a treatment be effective would influence the risk-taking preference [^1^](#_ENREF_1)^,^[^2^](#_ENREF_2).
2. For the ADE task, group RPI significantly differed between each condition of overall probability, when relative ΔP is smaller (Q_(3)_ = 49.2, P < 0.001) and when relative ΔP is larger (Q_(3)_ = 39.0, P < 0.001). Group RPI increased as the overall probability decreased (Fig. 3*B*, the middle panel). The results confirmed our previous findings that the overall probability that an adverse effect would occur would influence the risk-taking preference ^[2](#_ENREF_2)^.
3. For the TE task, group RPI significantly differed between each condition of overall probability, when relative ΔP is smaller (Q_(2)_ = 36.3, P < 0.001) and when relative ΔP is larger (Q_(3)_ = 34.7, P < 0.001). Group RPI increased as the days delayed for the treatment to reach its maximal effect decreased (Fig. 3*B*, the right panel). The results confirmed our previous findings that the time delay before the treatment reaches its maximal effect would influence the risk-taking preference ^[2](#_ENREF_2)^.
4. Between the scenarios of smaller relative ΔP and larger relative ΔP, we found a significant difference in group RPI in the ANE task (McNemar test, P = 0.004 when overall probability =90% and P = 0.003 when overall probability =50%). The findings are consistent with our previous findings [^1^](#_ENREF_1)^,^[^2^](#_ENREF_2), showing that the relative ΔP between the riskier and the riskless options would influence risk-taking preference.

References

1 Lin, C. Making the decision to stop pain: Probability and magnitude effects of expected pain relief on the choice of analgesics. *Eur J Pain* 17, 587-598, doi:10.1002/j.1532-2149.2012.00214.x (2013).

2 Lin, C. S., Wu, S. Y. & Wu, L. T. Preferences for Analgesic Treatments Are Influenced by Probability of the Occurrence of Adverse Effects and the Time to Reach Maximal Therapeutic Effects. *PloS one* 10, e0130214, doi:10.1371/journal.pone.0130214 (2015).

3 McNeil, D. W. & Rainwater, A. J., 3rd. Development of the Fear of Pain Questionnaire--III. *Journal of behavioral medicine* 21, 389-410 (1998).

4 Asmundson, G. J., Bovell, C. V., Carleton, R. N. & McWilliams, L. A. The Fear of Pain Questionnaire-Short Form (FPQ-SF): factorial validity and psychometric properties. *Pain* 134, 51-58, doi:10.1016/j.pain.2007.03.033 (2008).

5 Sullivan, M. J. L., Bishop, S. R. & Pivik, J. The Pain Catastrophizing Scale: Development and validation. *Psychological Assessment* 7, 9 (1995).

6 Spielberger, C. D., Gorsuch, R. L. & Lushene, R. E. *Test manual for the State Trait Anxiety Inventory*. (Consulting Psychologists Press, 1970).

7 Yarkoni, T., Poldrack, R. A., Nichols, T. E., Van Essen, D. C. & Wager, T. D. Large-scale automated synthesis of human functional neuroimaging data. *Nature methods* 8, 665-670, doi:10.1038/nmeth.1635 (2011).

8 Rubinov, M. & Sporns, O. Complex network measures of brain connectivity: uses and interpretations. *NeuroImage* 52, 1059-1069, doi:10.1016/j.neuroimage.2009.10.003 (2010).
